# Long-Term Follow-up of a Case with Proprotein Convertase 1/3 Deficiency: Transient Diabetes Mellitus with Intervening Diabetic Ketoacidosis During Growth Hormone Therapy

**DOI:** 10.4274/jcrpe.3986

**Published:** 2017-09-01

**Authors:** E. Nazlı Gönç, Alev Özön, Ayfer Alikaşifoğlu, Nurgün Kandemir

**Affiliations:** 1 Hacettepe University Faculty of Medicine, Department of Pediatric Endocrinology, Ankara, Turkey

**Keywords:** Proprotein convertase 1/3 deficiency, diabetes mellitus, diabetic ketoacidosis, treatment, diabetes insipidus

## Abstract

Proprotein convertase 1/3 (PC1/3) deficiency is a very rare disease characterized by severe intractable diarrhea in the first years of life, followed by obesity and several hormonal deficiencies later. Diabetes mellitus requiring insulin treatment and diabetic ketoacidosis have not been reported in this disorder. We herein present a girl with PC1/3 deficiency who has been followed from birth to 17 years of age. She developed deficiencies of all pituitary hormones over time as well as diabetes mellitus while receiving growth hormone (GH) therapy. She was complicated with diabetic ketoacidosis during dietary management of diabetes mellitus, thus insulin treatment was initiated. Insulin requirement to regulate hyperglycemia was short-lived. Repeat oral glucose tolerance test five years later was normal. The findings of this patient show that diabetes mellitus can develop at any time during follow-up of cases with proportein convertase 1/3 deficiency especially under GH therapy.

**What is already known on this topic?**

Diabetes mellitus may develop over time during the course of the disease, which may be due to insufficient conversion of proinsulin into insulin.

**What this study adds?**

Proprotein convertase 1/3 is an enzyme that converts prohormones into active hormones. Thus, proprotein convertase 1/3 deficiency has been reported to be characterized by several hormonal deficiencies. Elevation of proinsulin levels is used in the diagnosis; however, diabetes mellitus has not been reported before.

## INTRODUCTION

Proprotein convertase 1/3 (PC1/3) is an enzyme that is responsible for conversion of inactive peptides into active form. It is particularly expressed in neuroendocrine tissues. Thus, its deficiency leads to insufficient activation of several hormones including proinsulin, proopiomelanocortin, pro-thyrotropin-releasing hormone, pro-glucagon, and pro-gonadotropin-releasing hormone (1,2). To date, fewer than 20 patients with PC1/3 deficiency have been reported (3,4,5,6,7,8,9). Clinical presentation of these patients is variable. However, intestinal malabsorption in the first years of life and obesity thereafter are relatively constant findings. Other manifestations such as hypocortisolism, hypothyroidism, diabetes insipidus, hypogonadism, growth deficiency, and disorders of glucose metabolism are not seen in every patient. The time of onset for development of these hormone deficiencies is also variable (3,4,5,6,7,8,9).

Insulin deficiency due to inefficient conversion of proinsulin to insulin is one of the hallmarks of the disease. High proinsulin level is a diagnostic marker for PC1/3 deficiency. However, the patients reported so far did not have a significant disorder related to glucose metabolism.

Herein, we report a long-term follow-up of a 19-year-old girl with PC1/3 deficiency who developed multiple pituitary hormone deficiencies. She had a transient period of insulin-dependent diabetes mellitus with an intervening diabetic ketoacidosis during growth hormone (GH) therapy.

## CASE REPORT

A female proband was born by cesarean section with a birth weight of 3.5 kg. She was the only child of second-degree cousins of Turkish origin. Chronic diarrhea started in the first week of life. She was hospitalized several times for severe dehydration and metabolic acidosis. Total parenteral nutrition was started at 9 months of age and she was followed at a medical center for six months. Subsequently, the parents managed to offer parenteral nutrition to the patient in the household setting till she reached age 2 years. Since glucose, galactose, lactose, and long-chain fatty acids in the diet increased the amount and frequency of loose stools, they were eliminated from the oral feedings. The intestinal biopsy showed villous atrophy with nonspecific changes. Her appetite was so good that although the diarrheic attacks continued, the patient gained weight. During infections, attacks of metabolic acidosis reappeared, suggesting renal tubular acidosis, and bicarbonate therapy was started. In the following 2 years, diarrhea has nearly resolved, but the restricted diet was continued. At 4.3 years of age, the patient was referred to pediatric endocrinology for polyuria and polydipsia. The parents have been aware of her increased water intake since infancy, but they did not consider it a problem till the cessation of diarrhea. She used to drink 3-4 liters a day.

When the patient was 4.3-year-old, her height was 96 cm (3-10p), weight 22 kg (97p), and body mass index (BMI) was 23.9 kg/m^2^ (> 95p). Her physical examination was normal, and she did not have any dysmorphic features. The laboratory findings were as follows: hemoglobin (Hb): 12.2, hematocrit: 36, white blood cell: 7600, platelet: 270.000, glucose: 77 mg/dL, Na: 137 mEq/L, K: 4.8 mEq/L, Cl: 116 mEq/L, blood urea nitrogen: 4.1 mg/dL, creatinine 0.53 mg/dL, calcium: 9.9 mg/dL, P: 4.2 mg/dL, alkaline phosphatase: 373 U/L, alanine aminotransferase: 28 U/L, aspartate aminotransferase: 42 U/L. Blood gas analysis revealed: pH: 7.43 and bicarbonate (HCO_3_): 24.3 mmol/L. Urine density was 1003 and no proteinuria or glucosuria was noted. Water deprivation test yielded an increase in Na level to 151 mEq/L and a urine osmolality to 238 mOsmol/kg, while plasma osmolality was 320 mOsmol/kg. Simultaneous plasma arginine vasopressin (AVP) after water deprivation test was 1.3 pg/mL (0-8). Administration of intranasal DDAVP at a test dose of 5 µg increased the urine osmolality and alleviated the symptoms of polyuria and polydipsia. Diagnosis of central diabetes insipidus was established and intranasal DDAVP at a dose of 1.25 µg per day was started. At that time, morning cortisol, free thyroxine (fT_4_), and prolactin levels (cortisol: 19.8 µg/dL, fT_4_: 15. 2 pmol/L, prolactin: 8.2 ng/mL) were normal, insulin-like growth factor 1 (IGF-1) and IGF binding protein-3 (IGFBP-3) levels were low [IGF-1: 15 ng/mL (<-3 standard deviation [SD]) and IGFBP3: 1529 ng/mL (-2 SD–[-3 SD])]. Her weight and height gains are shown in Figures 1a and 1b.

At age 9 years, when her height was at 10^th^ percentile, fT_4_ was found to be lower than the normal range (fT_4_: 11.9 pmol/L, normal: 12-22; thyroid-stimulating hormone: 3.6 mIU/L, normal: 0.27-4.2). Fasting morning cortisol level was 4 µg/dL and adrenocorticotropic hormone (ACTH) was 21 pg/mL. Low-dose ACTH test was performed and cortisol peak was subnormal at 15 µg/dL [N: 19.8 µg/dL] (10). The diagnosis of central hypothyroidism and adrenal insufficiency were established, and Na L-thyroxin (100 µg per day) and hydrocortisone (10 mg/m^2^ per day in three doses) replacements were started accordingly.

At the age of 10.5 years, she was 129.7 cm in height [-1.49 standard deviation score (SDS)], and growth velocity decreased to 1.8 cm/year (Figures 1a, 1b). Her bone age was 8 years. The midparental height was 156.25 cm (-0.99 SDS). The levels of IGF-1 and IGFBP3 were 56 ng/mL (<-3 SD) and 1848 ng/mL (-3 SD), respectively. GH stimulation tests with levodopa and clonidine were carried out and peak GH responses were 4.03 and 4.6 ng/mL, respectively (normal GH response: >7 ng/mL). Recombinant human GH (rhGH) was started at a dose of 0.03 mg/kg per day subcutaneously. The repeat magnetic resonance imaging of the pituitary gland was normal with a 6-mm height in the anterior lobe and a normal bright spot on the posterior lobe.

At the age of 11.5 years, after receiving GH therapy for one year, the patient had gained 7 cm. Her height was 134.5 cm and weight 50.3 kg, with a BMI 27.9 kg/m^2^ (>97p). The diagnosis of PC1/3 deficiency was established by the mutation analysis of PCSK1 gene. A novel essential splice site mutation (IVS8+1G>T) was identified (7).

**Figure 2:**
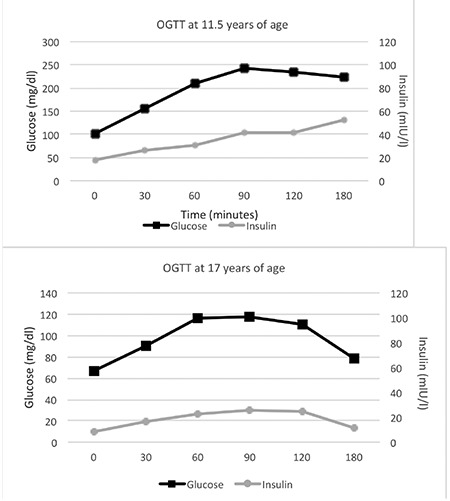
Oral glucose tolerance test at two different time points (11.5 and 17 years of age) OGTT: oral glucose tolerance test

Fasting blood glucose level was 85 mg/dL, and there were neither signs and symptoms nor any family history for diabetes mellitus. However, a derangement in glucose metabolism was likely in PC1/3 deficiency, so oral glucose tolerance test was performed. The results revealed diabetes mellitus (Figure 2). HbA1c was 5.8% (4.5-6.2). Anti-insulin, anti-GAD, and anti-IA2 antibodies were negative. Weight loss, physical activity, and diabetic diet were recommended. GH therapy was not discontinued.

Three months later, HbA1c increased to 6% and continued to increase to 6.5% in the next 6 months on GH treatment. At the age of 12.5 years, she was brought to emergency clinic by her parents for lethargy. No signs or symptoms of infection were noted. She was dehydrated. Blood glucose was 725 mg/dL (simultaneous insulin level was 15 mIU/L) with ketosis (urine ketones were 4+), and acidosis (blood pH: 7.15 and HCO_3_: 9.2 mmol/L). HbA1c was 10.5%. Diabetic ketoacidosis was treated with intravenous fluid-electrolyte and insulin therapy. Basal-bolus insulin regimen using rapid-acting insulin three times a day and long-acting insulin, glargine, once a day was started thereafter. Initially, total daily dose of insulin was nearly 1.5 U/kg. However, the daily requirement of insulin progressively decreased to 0.15 U/kg per day within 10 days and eventually it was discontinued within one month. HbA1c levels were between 5.3 and 6.2% and fasting and postprandial glucose levels remained within normal levels thereafter. She received GH treatment till 15.1 years of age under a diabetic diet without any further deterioration in glucose metabolism.

The patient remained prepubertal during her follow-up and at 13 years of age, gonadotropin and estradiol levels were very low (follicle-stimulating hormone<0.07 mIU/mL, luteinizing hormone<0.07 mIU/mL, E2: 2.65 pg/mL), so estradiol replacement was started at the age of 13 years and switched to cyclic treatment at 15.5 years.

At 16.5 years, her final height is 153 cm (-1.5 SDS) and weight 69 kg (body mass index: 29.5 kg/m2). She is receiving Na l-thyroxine (2 mcg/kg/day) for hypothyroidism, sublingual lyophilized DDAVP tablet (30 µg two times a day) for central diabetes insipidus, hydrocortisone (10 mg/m^2^/day) for adrenal insufficiency, combined estrogen-progesterone pills for hypogonadism, and a diabetic diet for diabetes mellitus.

At the age of 17 years, HbA1c was 5.4% and a repeat oral glucose tolerance test showed normal glucose homeostasis (Figure 2).

## DISCUSSION

Multiple hormonal insufficiencies have been reported in patients with PC1/3 deficiency. However, every patient with PC1/3 deficiency varies in the nature of hormonal insufficiency as well as its severity. The first reported case with PC1/3 deficiency was a 43-year-old woman who had obesity, hypogonadotropic hypogonadism and hypoadrenalism (3,11). Although GH response to insulin-induced hypoglycemia was low, she had a normal height of 161 cm. Oral glucose tolerance test showed an elevated two-hour blood glucose level (206 mg/dL) indicating diabetes mellitus. She also had postprandial hypoglycemia after a standardized meal (3).

The second patient was a female infant with intractable diarrhea who subsequently developed obesity (4). She had several episodes of hypoglycemia which were attributed to low cortisol response to hypoglycemia. Hydrocortisone replacement was started. She died of uncertain cause at the age of 18 months (4).

The third case was a boy who was followed till six years of age (5). He developed severe obesity after a period of intractable diarrhea which required 5-week parenteral nutrition in addition to oral feedings with specialized infant formula. At the age of 4 years, he developed polyuria and polydipsia. However, the water deprivation test was not diagnostic for diabetes insipidus. Consequently, following the diagnosis of PC1/3 deficiency, he was further evaluated for hormone insufficiencies; hypocortisolism and hypothyroidism were detected. In the case report, there was no detailed information about glucose metabolism of the boy except a normal fasting glucose and elevated proinsulin levels (5).

The fourth case was the first report in the literature with PC1/3 deficiency who had a documented central diabetes insipidus (6). He had hypocortisolism, hypothyroidism, and low testosterone level with micropenis suggesting hypogonadism as well. He had a normal GH response at the time of hypoglycemia. No further evaluation of glucose metabolism was mentioned in the report (6).

Then, Martín et al (7) reported the clinical, laboratory, and genetic features of 13 children with PC1/3 deficiency from 11 families. Our patient was in that cohort (represented as family 3). Eleven of thirteen cases reported by Martín et al (7) were alive and 8 were younger than 10 years old. Hypothyroidism, hypocortisolism, and diabetes insipidus were relatively more common than GH deficiency in that cohort. It was reported that the patients who received GH had had a good response. Data about glucose metabolism of the patients was scarce except a note of postprandial hypoglycemia in 8 of the cases. Oral glucose tolerance test or HbA1c levels were not determined.

We had the opportunity to follow our patient from birth to 17 years of age and to observe nearly all consequences of PC1/3 deficiency reported so far. PC1/3 activity is essential for the activating cleavage of many peptide hormone precursors including hypothalamic hormones (1,2). So, lack of activation of hypothalamic hormones may mimic multiple pituitary hormone deficiency due to a defect in hypothalamus-pituitary axis. Diabetes insipidus was the earliest hormonal deficiency detected in the current patient. It probably started even before, possibly early in infancy but was disregarded till 4 years of age as the parents assigned the symptoms of polyuria and polydipsia to ongoing diarrhea.

Thyroid hormones and cortisol level were in normal ranges till 9 years of age in the current patient. Although IGF-1 and IGFBP-3 levels were low at 4 years of age, height was at the 10^th^ percentile and growth velocity was normal. At 10.5 years, growth velocity decreased, GH response was low in GH stimulation tests and rhGH was initiated at a conventional dose. One year after the GH therapy, the diagnosis of PC1/3 deficiency was established definitively by genetic analysis. Oral glucose tolerance test was performed since a potential disorder in glucose metabolism was considered and diabetes mellitus was diagnosed. Insulin response to elevated glucose levels indicated neither absolute insulinopenia nor insulin resistance, however suggested a relative insulin deficiency. There was no symptom suggestive of hyperglycemia at the time of testing. HbA1c increased to 6.5% while the patient was on a diabetic diet, and two years after the onset of rhGH therapy (at age 12.5 years), diabetic ketoacidosis developed without any identifiable precipitating cause. Insulin requirement continued for one month only. Although rhGH therapy was continued, a similar picture of insulin insufficiency did not recur till 15.1 years of age. Two years after cessation of rhGH therapy, a repeat oral glucose tolerance test was completely normal.

Diabetes mellitus was not defined as a part of PC1/3 deficiency although it can be speculated that there must be a relative insulin deficiency due to the defect in conversion of proinsulin to insulin. The patients reported so far did not have history of low birth weight suggesting insulinopenia during intrauterine life. Diabetes mellitus was identified only in the first reported patient with PC1/3 deficiency (3). She developed gestational diabetes mellitus requiring insulin treatment (3). The same patient was tested again at age 43 years and at that time, her 2-hour post-load blood glucose level was 206 mg/dL (3).

Our patient is the first patient with PC1/3 deficiency who developed diabetic ketoacidosis. Diabetic ketoacidosis and one month of insulin requirement coincided with rhGH therapy which may be a contributing factor for relative insulin deficiency due to the anti-insulin effect of GH (12). However, since the insulin requirement was transient even in the course of rhGH therapy in our patient, it is difficult to consider GH as the sole factor responsible for deterioration of glucose metabolism. Diabetic ketoacidosis can complicate cases with excess GH secretion such as gigantism or acromegaly (13,14,15). However, we found only one report of a patient developing diabetic ketoacidosis during GH therapy (16). The case was a 13-year-old boy with Prader-Willi syndrome who presented with diabetic ketoacidosis four weeks after initiation of GH treatment (16). The status of glucose metabolism before GH was unknown in this patient and hyperglycemia resolved just 2 months after cessation of GH treatment. Later, this boy was diagnosed as type 2 diabetes (16). Thus, impaired glucose metabolism can associate with GH treatment, but diabetic ketoacidosis is very unlikely to develop and in such a case, presence of a predisposing condition needs to be investigated. Previous reports of patients with PC1/3 deficiency do not include details of routine investigations of glucose homeostasis, especially glucose tolerance test. The most commonly reported disturbance in glucose metabolism was postprandial hypoglycemia. Therefore, the true prevalence of diabetes mellitus in cases with PC1/3 deficiency is yet unknown.

Disorders of glucose homeostasis should be assessed in patients with PC1/3 deficiency. Diabetes mellitus with asymptomatic hyperglycemia may be one of the disorders of hormone metabolism in PC1/3 deficiency. There may be periods with relative or sometimes even severe deficiency of insulin (i.e. leading to ketoacidosis) requiring insulin treatment especially under GH treatment.

## Figures and Tables

**Figure 1a:**
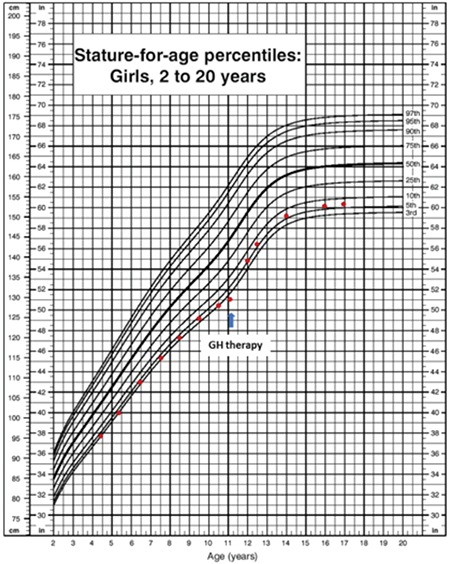
Growth chart of the patient before and after growth hormone therapy GH: growth hormone

**Figure 1b:**
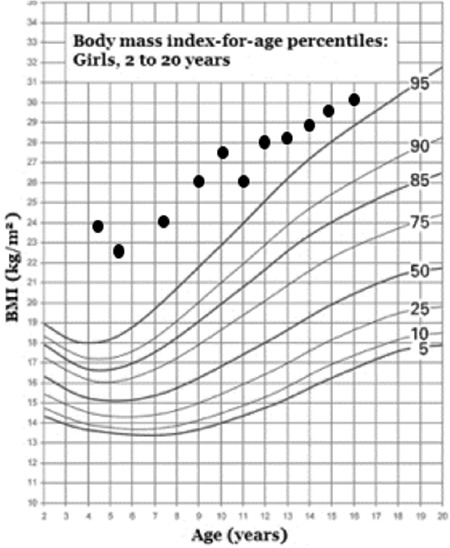
Body mass index chart of the patient BMI: body mass index
